# „Generationen in der Medizin“

**DOI:** 10.1007/s10354-023-01005-9

**Published:** 2023-03-29

**Authors:** Anahit Anvari-Pirsch

**Affiliations:** Teaching Center und Curriculum Humanmedizin, Medizinische Universität Wien, Spitalgasse 23/BT 87, 1090 Wien, Österreich

„Es ist das Schicksal jeder Generation, in einer Welt unter Bedingungen leben zu müssen, die sie nicht geschaffen hat.“ (John. F. Kennedy)

Die Bezeichnungen *Generation X, Y* bzw. *Z* beschreiben jeweils verschiedene Altersgruppen, wobei sich deren Zugehörige durch bestimmte, in der jeweiligen Kohorte verstärkt auftretende, Eigenschaften und Fähigkeiten auszeichnen. Ausgehend von den Sozialwissenschaften, der Marketingforschung und den Human Resources fanden die Generationenbezeichnungen und die damit zusammenhängenden konzeptionellen Vorstellungen Eingang in Populärkultur und Alltagssprache.

Grundsätzlich stellt der Versuch, mittels pauschalisierender Aussagen bestimmten Personengruppen konkrete Eigenschaften zuzuschreiben, stets ein, mit einer gewissen Unschärfe einhergehendes, Wagnis dar. Dies gilt gleichsam für die Kategorisierung von Ärzt:innen – etwa im Hinblick auf Ausbildungswege, Erwartungen an den Beruf sowie die jeweilige professionelle Haltung – anhand der Generationenzugehörigkeit. So verfügt jede:r Einzelne über individuelle Erfahrungen und Einstellungen, wobei sich ihre:seine persönlichen Herangehensweisen deutlich voneinander unterscheiden können. Zugleich lassen sich bestimmte Tendenzen, die in den jeweiligen Generationen von X bis Z vermehrt festgestellt werden können, nicht von der Hand weisen, weshalb eine Betrachtung aus der Generationenperspektive durchaus zu einem klareren Bild beitragen kann.

Die Generation X umfasst jene Personen, die zwischen den mittleren 1960er- und 1980er-Jahren geboren wurden. Diese Alterskohorte trägt den Beinamen *Sandwich-Generation*, welcher auf die klassische Doppelbelastung der Generation X referenziert, die sich aus dem Umstand speist, dass sie häufig sowohl für die finanzielle Unterstützung der eigenen Kinder (durch deren oft bis ins fortgeschrittene Erwachsenenalter hinein andauernde Studienzeit) als auch für die Betreuung älterer Familienmitglieder verantwortlich sind. Letzteres gilt insbesondere für Frauen. Weiters sind die Angehörigen dieser Generation durch ein starkes Streben nach Unabhängigkeit sowie eine ausgeprägte Anpassungsfähigkeit – vor allem im Vergleich zu der vorhergehenden *Baby-Boomer-Generation* (Stichwort: technologische Entwicklung) – charakterisiert. Ihre medizinische Ausbildung haben sie zum Großteil zu einer Zeit absolviert, als das Internet noch nicht omnipräsent war und die digitale Revolution erst langsam an Fahrt aufnahm. Didaktisch orientierte sich die Ausbildung daher vorrangig an traditionellen Lehrmethoden, wie zum Beispiel klassischen Hörsaal-Vorlesungen und Hands-on-Praktika. Zugleich war die Arbeitswelt zur Zeit des Berufseintrittes der Generation X vergleichsweise stabil und sicher. Viele Menschen dieser Generation schlugen daher traditionelle Ausbildungs- und Karrierewege ein. Entsprechend ihrer Erfahrung vertreten sie die Auffassung, dass eine gute Ausbildung den Schlüssel zu beruflichem Erfolg darstellt. Als Mediziner:innen steht für sie die Patient:innenbeziehung im Vordergrund, während technologische Hilfsmittel tendenziell zweitrangig sind. Angenommen wird daher eine stärkere Fokussierung der Generation X auf die menschliche Seite der Medizin sowie eine gewisse Skepsis gegenüber technologischen Innovationen.

Die *Generation Y*, deren Mitglieder auch als *Millenials* bekannt sind, umfasst all jene Personen, die zwischen den frühen 1980er- und späten 1990er-Jahren geboren wurden. Millenials sind großteils mit der Digitalisierung aufgewachsen – wenngleich vor allem die älteren dieser Gruppe keine *Digital Natives* per se darstellen – und haben daher ein vertrautes Verhältnis zu modernen Kommunikationstechnologien und Social Media. Sie sind oft selbstbewusst und wollen in ihrem Beruf erfolgreich sein, wobei sich dieser Erfolg nicht mehr rein in prestigeträchtigen Positionen und lukrativen Gehältern bemessen lässt: Die Work-Life-Balance ist für die Generation Y von großer Bedeutung. Die medizinische Ausbildung absolvierten sie bereits vor dem Hintergrund einer stark verbreiteten Internetnutzung. Im Zuge der digitalen Revolution hielten technologische Innovationen Einzug in den Ausbildungsalltag. Dazu zählen beispielsweise E‑Learning-Plattformen. Im gleichen Atemzug gestaltete sich die Arbeitswelt allerdings zunehmend schnelllebig und unsicher. Viele Menschen dieser Generation haben daher eine flexiblere Haltung gegenüber Karriere und Ausbildung und zeigen, im Vergleich zur Generation X, ein geringeres Interesse an traditionellen Karrierewegen. Stattdessen steht eine Ausbildung im Zentrum, die es ihnen ermöglicht, ihre Talente zu fördern und zugleich die eigenen Interessen zu entfalten. Sie weisen meist eine größere Affinität zu digitalen Werkzeugen auf und scheinen eher bereit zu sein, technologische Innovationen in den beruflichen Alltag zu integrieren sowie datenbasierte Entscheidungen zu treffen.

Die *Generation Z*, deren Angehörige auch als *Zoomer* bezeichnet werden, umfasst ungefähr die ersten zehn bis fünfzehn Jahrgänge ab der Jahrtausendwende. Diese Generation ist die erste, die vollständig in der digitalen Welt aufgewachsen ist, weshalb sie eine, im Vergleich mit den Generationen X und Y, noch engere Bindung zu digitalen Technologien und Social Media aufweist. Die Generation Z absolviert ihre medizinische Ausbildung in einer stark von der digitalen Revolution geprägten Welt. In Bezug auf die Arbeitswelt sieht sich die Generation Z stark ausgeprägten Änderungsdynamiken und Unsicherheiten gegenüber. Dies führt unter anderem dazu, dass sie hinsichtlich Karriere und Ausbildung als noch flexibler gelten. Als Ärzt:innen wird der Großteil der Zoomer erst in den nächsten Jahren in die Berufspraxis einsteigen. Anzunehmen ist ein intuitiver Umgang mit Technologie und Datenanalyse und damit einhergehend eine stärkere Nutzung dieser Möglichkeiten für medizinische Zwecke. Außerdem gilt die Generation Z, im Vergleich zu X und Y, als deutlich idealistischer, was sich auch in der Alternativbezeichnung *Generation Greta* (Anm.: in Anlehnung an die *Fridays-For-Future*-Gründerin Greta Thunberg) widerspiegelt. Hinzukommen ein großer Selbstwert und Individualismus. Dementsprechend sind sie weniger an der klassischen Karriere als einer sinnstiftenden sowie die individuellen Bedürfnisse befriedigenden Tätigkeit interessiert. Zugleich sind Zoomer alleine aufgrund des demographischen Wandels begehrte Arbeitskräfte, die gerade auch im Gesundheitssektor einem wachsenden Bedarf gegenüberstehen.

Eine generationale Betrachtungsweise kann hilfreich sein – wobei jedoch berücksichtigt werden sollte, dass die medizinische Ausbildung auf globaler Ebene unterschiedlich gestaltet sein kann sowie selbstverständlich auch innerhalb einer Generation individuelle Unterschiede möglich sind. In Bezug auf Haltung und Erwartung der (zukünftigen) Ärzt:innen spielen – neben der Zugehörigkeit zu einer bestimmten Generation – eine Vielzahl an weiteren Faktoren, etwa persönliche und soziokulturelle Werte sowie individuelle Erfahrungen, eine große Rolle.

Nicht zuletzt gilt: Jede Generation verfügt über ihre jeweiligen Stärken und Schwächen, weshalb es keine *bessere* oder *schlechtere* Generation gibt. Jede Alterskohorte hat ihren eigenen Einfluss auf die Welt und trägt auf ihre spezielle Weise zur Vielfalt und Dynamik unserer Gesellschaft bei.
